# Structural Analysis of the Outer Membrane Lipoprotein BBA14 (OrfD) and the Corresponding Paralogous Gene Family 143 (PFam143) from *Borrelia burgdorferi*

**DOI:** 10.3390/pathogens11020154

**Published:** 2022-01-26

**Authors:** Inara Akopjana, Kalvis Brangulis

**Affiliations:** Latvian Biomedical Research and Study Centre, LV-1067 Riga, Latvia; inara@biomed.lu.lv

**Keywords:** Lyme borreliosis, *Ixodes* ticks, spirochetes, X-ray crystallography

## Abstract

Lyme disease is caused by the spirochete *Borrelia burgdorferi,* which can be transmitted to a mammalian host when infected *Ixodes* ticks feed. *B. burgdorferi* has many unique characteristics, such as the presence of at least 130 different lipoproteins, which is considerably more than any other known bacterium. Moreover, the *B. burgdorferi* genome is relatively small (1.5 Mbp) but at the same time it is quite complicated because it comprises a chromosome and 21 linear and circular plasmids. *B. burgdorferi* is also rich in paralogous proteins; in total, there are approximately 150 paralogous gene families. Equally important is the fact that there is still no vaccine against the Lyme disease. To better understand the role of lipoproteins in this unique bacterium, we solved the crystal structure of the outer membrane lipoprotein BBA14, which is coded on the relatively stable linear plasmid 54 (lp54). BBA14 does not share sequence identity with any other known proteins, and it is one of the ten members of the paralogous gene family 143 (PFam143). PFam143 members are known as orfD proteins from a genetic locus, designated 2.9. The obtained crystal structure revealed similarity to the antitoxin from the epsilon/zeta toxin-antitoxin system. The results of this study help to characterize BBA14 and to clarify the role of PFam143 in the lifecycle of *B. burgdorferi*.

## 1. Introduction

Lyme disease is an increasingly common infectious disease and is caused by the spirochete *Borrelia burgdorferi* sensu lato complex bacteria that includes *B. burgdorferi* sensu stricto (hereafter *B. burgdorferi*), *B. bavariensis*, *B. afzelii*, *B. garinii*, *B. mayonii,* and *B. spielmanii* [1,2,3,4,5,6]. The spirochetes can enter the mammalian host during the blood meal of an infected *Ixodes* tick [7,8].

*B. burgdorferi* has several unique features that are different from other bacteria. First, the existence of a large number of extrachromosomal DNA elements in addition to the chromosome (12 linear and 9 circular plasmids) [9,10,11]. It should be noted that not all plasmids and their encoded proteins are equally required at different stages of the bacterial lifecycle because continuous in vitro cultivation of *B. burgdorferi* results in the loss of some plasmids and the loss of infectivity in animal models [12,13]. Although some of the plasmids, for example, lp54 and cp26, have been found to be evolutionary and structurally stable components of the *B. burgdorferi* genome, the presence of most other circular and linear plasmids can differ between low- and high-infectivity clones, for example, plasmids lp25 and lp28-1 have been recognized as infectivity-associated plasmids because they are absent only in low-infectivity clones, while plasmids lp56 and cp9 are not required for infectivity in C3H/HeN mice [11,14,15]. Second, an exceptionally high pseudogene content—in some linear plasmids the damaged gene content can reach up to 50% [9,10]. Third, a high number of paralogous genes, since a large portion of *B. burgdorferi* genes contain at least one additional copy; thus, there are approximately 150 paralogous gene families (PFams). A small number of paralogous genes are found on the chromosome but the majority are located on the 9 circular or 12 linear plasmids, and overall, most of the genes coded on the plasmids are members of paralogous families [9,10,11]. Fourth, a lack of any similarity to genes in other organisms. Approximately 90% of plasmid coding genes do not show resemblance with those in other organisms [9,10]. Fifth, an atypical number of lipoproteins. *B. burgdorferi* contains at least 130 different lipoproteins, i.e., proteins that contain an N-terminal signal sequence containing a four amino acid motif, known as a lipidation consensus sequence or lipobox, followed by a mandatory Cys residue where the fatty acids from the membrane bilayer are covalently attached and which becomes the new N-terminal residue after proteolytic removal of the signal peptide [9,16,17]. Given that these surface lipoproteins are in direct contact with the environment, it is not surprising that some lipoproteins have already been associated with a variety of processes important for the pathogenesis, such as fight against the host’s immune response [18,19,20], tick-to-vertebrate transmission [21,22,23,24] and dissemination [25,26]. Considering the specific life cycle of this bacterium and that it is exposed to different environmental conditions to which it must be able to adapt, it is no wonder that *B. burgdorferi* has so many surface lipoproteins [27].

Because of these features, it is important to study *B. burgdorferi* to understand the mentioned differences of this bacterium. In the current study, we focused on the previously poorly characterized lipoprotein BBA14, which is known to be highly immunogenic protein recognized by sera from Lyme disease patients [28], but which does not have sequence similarity to any other known proteins in other organisms and is one of the 10 members of PFam143. Only a few of the paralogous gene families of *B. burgdorferi*, such as PFam54_60, have been comprehensively characterized and the molecular and functional details have been revealed for at least a single member [29]. In the current study, 3D structural data and a sequence analysis elucidated details of BBA14 and this information helps to outline new research directions for this paralogous gene family to fully understand its role in the lifecycle of *B. burgdorferi*. By studying this protein in more detail and due to the fact that BBA14 is located on the surface of the bacterium, it cannot be ruled out that in the future it could be also tested as a vaccine candidate.

## 2. Results and Discussion

### 2.1. Crystal Structure of B. burgdorferi BBA14

**Figure 1:**
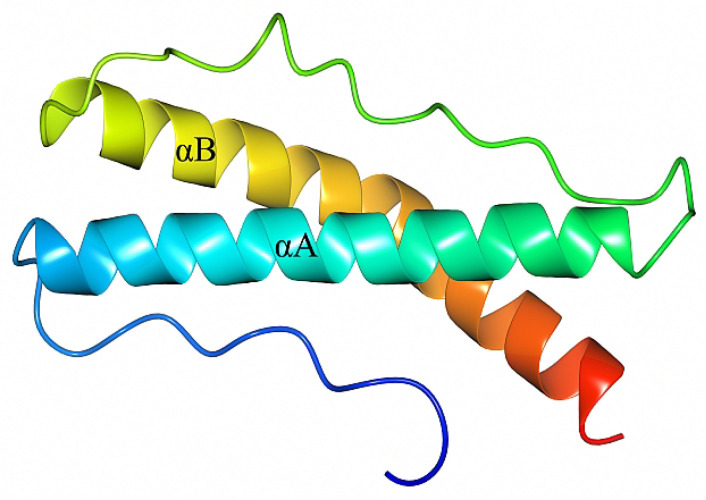
Crystal structure of *B. burgdorferi* BBA14_26–118_ (PDB ID 7QDV). The structure is colored from blue at the N-terminus to red at the C-terminus and includes the extra N-terminal residues Ala-Met-Gly from the expression tag. The α-helices are labeled as αA and αB.

Crystals of BBA14 were obtained by using the recombinant protein BBA14_26–121_, which corresponds to the full-length protein except the N-terminal signal peptide. The crystals were in space group P4_1_2_1_2, with one molecule per asymmetric unit. In the obtained crystal structure, the last three C-terminal residues 119–121 were not modeled due to weak electron density, while at the N-terminus, also the residues Ala-Met-Gly remaining after TEV protease digestion were modeled. The fold of BBA14 is formed by two α-helices, designated αA and αB, comprising 27 and 30 residues, respectively. The α-helices are connected by a 20 amino acid loop region; thus, αA and αB are placed in parallel (Figure 1). Additionally, there is an N-terminal loop region comprising 15 amino acids; given that BBA14 is a lipoprotein, this loop likely serves as a flexible region that connects the folded region to the cell membrane.

### 2.2. BBA14 as a Member of the Paralogous Gene Family 143

**Figure 2:**
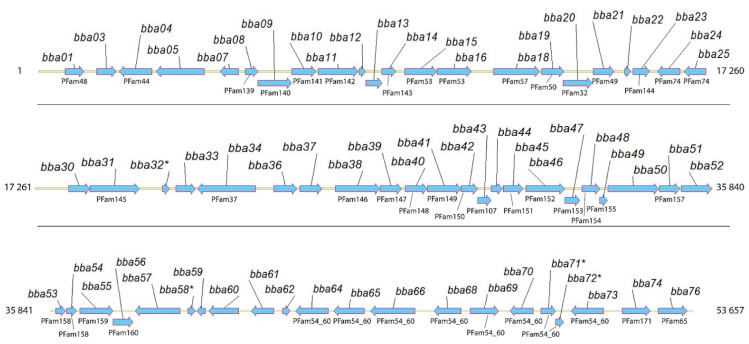
Full-length *B. burgdorferi* lp54 coding region corresponding to *bba01*-*bba76*. For those genes for which paralogs have been found, the corresponding PFam is indicated. Potential pseudogenes are indicated with an asterisk.

*Bba14* is one among 65 intact protein-coding genes and a few pseudogenes found on lp54—a linear plasmid known to carry many essential virulence factors such as BBA52, BBA57, BBA64, and BBA15 (also known as OspA) based on which the only human vaccine against Lyme disease was made available in 1998, although the vaccine was withdrawn from the market a few years later [22,23,25,30] (Figure 2).

**Figure 3:**
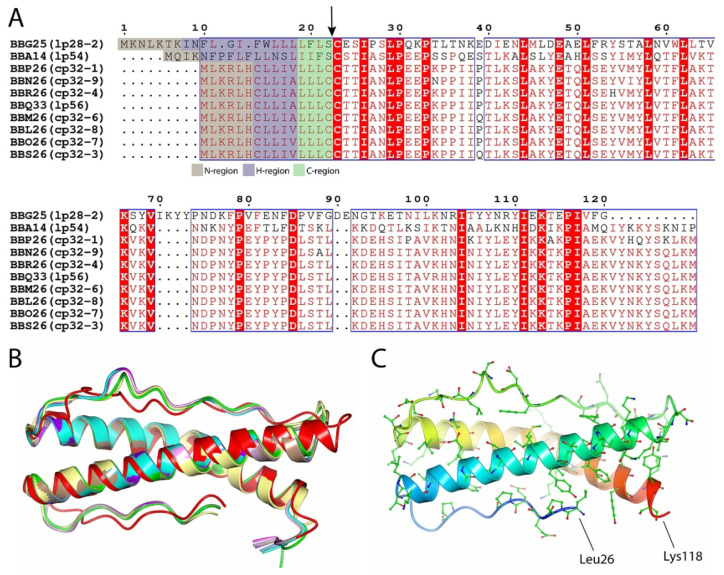
(**A**) Sequence alignment of *B. burgdorferi* PFam143 member proteins BBG25, BBA14, BBP26, BBN26, BBR26, BBQ33, BBM26, BBL26, BBO26, and BBS26. Sequence alignment was performed by using the Clustal Omega multiple sequence alignment tool and was further processed by ESPript 3 [31,32]. Conserved substitutions found between at least seven members used in the alignment are illustrated in red and framed, while the residues found to be identical between all of the PFam143 members are illustrated with a red background. The potential lipoprotein signal peptide cleavage site is indicated with an arrow, and the prediction of lipoprotein signal sequence regions is color coded, as indicated below the alignment. The numbering is illustrated for BBG25. For every member, the corresponding plasmid on which it is located is given in brackets. (**B**) Crystal structure of *B. burgdorferi* BBA14 (yellow, PDB ID 7QDV) superimposed with protein structures predicted with AlphaFold—BBG25 (red), BBA14 (green), BBP26 (cyan), BBN26 (pink), BBR26 (brown), BBQ33 (purple), BBM26 (gray), BBL26 (blue), BBO26 (magenta) and BBS26 (lilac). The N-terminal signal sequence region (hydrophobic α-helix) was excluded from the predicted protein structures. (**C**) Conserved residues between BBA14 and PFam143 members other than BBG25. The first and the last residues in BBA14 have been designated. A rainbow color scheme was used starting from blue at the N-terminus and gradually switching to red toward the C-terminus.

Initially, based on the full-genome sequence of *B. burgdorferi* and the subsequent data analysis, BBA14 was categorized as one of the 10 members of PFam143 (BBG25, BBA14, BBP26, BBN26, BBR26, BBQ33, BBM26, BBL26, BBO26, and BBS26) [9]. However, the status of PFam143 member BBA14 in the NCBI Gene and KEGG databases changed several times from a protein-coding gene to a pseudogene and vice versa. On one occasion also at our initiative the status in the NCBI Gene database was changed from a pseudogene to a protein-coding gene (ticket #28045-263315). At the time of manuscript preparation *bba14* in the NCBI Gene database is designated as a protein-coding gene (*bb_rs05175*), the same as other PFam143 members, although in the KEGG database *bba14* (bbu:BB_A14) is still designated as a pseudogene unlike the other PFam143 members. A sequence comparison revealed that the identity of the PFam143 members that are distributed over 10 different extrachromosomal elements varies from 25% to 99%, and with very slight differences, all of the proteins have the same length (Figure 3A). The PFam143 members BBP26, BBN26, BBR26, BBQ33, BBM26, BBL26, BBO26, and BBS26 share, on average, 98% mutual sequence identity and members BBL26 and BBO26 are completely identical. In turn, BBG25 and BBA14 are more distant family members because BBG25 shows, on average, 25% identity with the other PFam143 members but BBA14 has 46% identity.

Although BBA14, and especially BBG25, show relatively low sequence similarity to other PFam143 members, the PFam143 protein structure prediction with AlphaFold [33] indicated a highly conserved overall fold (C^α^ root-mean-square deviation from 0.87 to 1.63 Ǻ), where BBG25 showed the greatest difference from the overall fold (Figure 3B). In addition, the predicted structure of BBA14 (all the predictions were done before the crystal structure of BBA14 was deposited in the Protein Data Bank (PDB)) corresponded very well to the crystal structure of BBA14 (C^α^ root-mean-square deviation of 0.63 Ǻ). Given that the overall protein fold is conserved, it can be concluded that in BBG25, the conserved residues mainly represent the hydrophobic residues that are most likely involved in the overall fold preservation, with only a few conserved surface-exposed residues that are potentially related to the provision of the common function, if still preserved between the members. In turn, by indicating the conserved residues between BBA14 and the PFam143 members other than BBG25, the many surface-conserved residues strongly suggest a possible conservation of the function among these members (Figure 3C).

The PFam143 members are also known as OrfD proteins and are a part of a previously described genetic locus, designated as 2.9, and consisting of an operon of four genes *ABCD* and several adjacent lipoprotein coding genes [34]. Components *orfA* and *orfB* from the operon were found to code for a prophage-encoded holing-like system and were designated as BlyA and BlyB, respectively [35]. Although it has been clarified that OrfA is a membrane protein holin and OrfB is a soluble regulatory factor, the roles of *orfC* and *orfD* components have not been established. Interestingly, the operon *ABCD* was not previously attributed to lp54 but only to cp32 and lp56 [34,36], although sequence analysis indicated that BBA12 shares 30% identity with BBP23 (OrfA) and that BBA13 has 25% identity with BBP24 (OrfB) from cp32-1. Moreover, the AlphaFold protein structure prediction for BBA12, BBA13, BBP23, and BBP24 reveals that proteins BBA12/BBP23 and BBA13/BBP24 show similar overall protein folds (C^α^ root-mean-square deviation 1.88 Ǻ and 2.63 Ǻ respectively). However, due to the low sequence similarity, it is unclear whether BBA12 and BBA13 retain the same function as the corresponding paralogous proteins BBP23 (representing PFam109 consisting of 8 members) and BBP24 (representing PFam111 consisting of 8 members). In these two paralogous gene families, the members are identical or share nearly 100% sequence identity. Meanwhile, the component OrfC initially found as a part of the operon *ABCD* located on cp32 and lp56 and known as a PFam112 member was not found on lp54, since there are no other coding segments between BBA13 and BBA14 (Figure 2).

Spirochetes are characterized by the fact that the C-terminal region of a signal peptide, known as a lipobox, is relatively variable and often makes it difficult to predict borrelial lipoproteins [16]. Based on the initial analysis of the lipobox sequence it was suggested that in PFam143, only BBA14 and BBG25 are lipoproteins [9]. According to the latest algorithm designed for the identification of spirochaetal lipoproteins, the lipoprotein signal peptide is divided into the N-region (at least 2 residues long and up to the last charged residue), H-region (at least 6 residues long, hydrophobic, and does not contain charged residues), and C-region or lipobox (sequence of 4 specific residues followed by Cys, where Cys serves as the new terminal end after cleavage of the signal peptide) [16]. Based on the new algorithm, it can be concluded that the problem is not related to the C-region or lipobox of the signal peptide but to the fact that there is a deviation in the H-region, since it is only 5 residues in length and the last residue in the H-region must be Leu, Ile, Val, Phe, Tyr, or Met, which is not the case for all of the PFam143 members except BBA14 and BBG25 (Figure 3A). Localization studies for BBA14 and BBG25 have indicated that BBA14 is surface exposed, but BBG25 is most likely attached to the outer membrane and faces the periplasm [37]. The fact that the other PFam143 members are not secreted and lipidated was previously confirmed by observing the lack of lipidation or processing in *E. coli* [34]. However, although they are not lipidated, it is clear that the other PFam143 members still have a distinctive hydrophobic N-terminal α-helix that can serve as a transmembrane region for intracellular localization which also has been noted previously [34].

### 2.3. Structural Similarity

**Figure 4:**
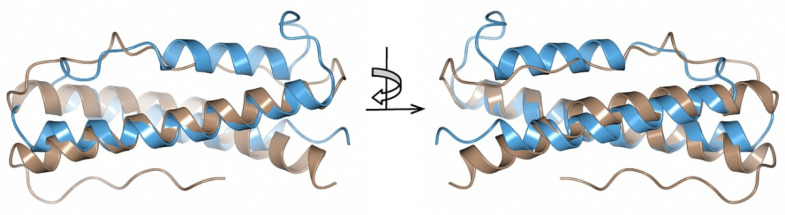
Superimposed crystal structures of *B. burgdorferi* BBA14 (brown, PDB ID 7QDV) and *S. pyogenes* antitoxin epsilon (blue, PDB ID 3Q8X). The crystal structures are shown at two different angles rotated by 180°.

By comparing with the available 3D protein structures deposited in the Protein Data Bank (PDB) using PDBeFold [38], the crystal structure of BBA14 revealed a structural similarity with the antitoxin epsilon from the toxin-antitoxin system in *Streptococcus pyogenes* [39,40] (Figure 4).

The toxin-antitoxin system is a common mechanism used by many bacteria to ensure stable plasmid maintenance. Although there are several classes of toxin-antitoxin systems based on the nature of interacting molecules and the mechanism of the toxic effect (there are more than 30 different toxin-antitoxin systems in *E. coli* alone), it usually requires two gene products, referred to as toxin and antitoxin, that are capable of forming a complex [41]. If antitoxin formation is stopped and the antitoxin is proteolytically degraded, the toxin can cause different destructive effects; for example, in *S. pyogenes,* the toxin inhibits the biosynthesis of the cell wall (peptidoglycan), thus provoking cell autolysis [39].

**Figure 5:**
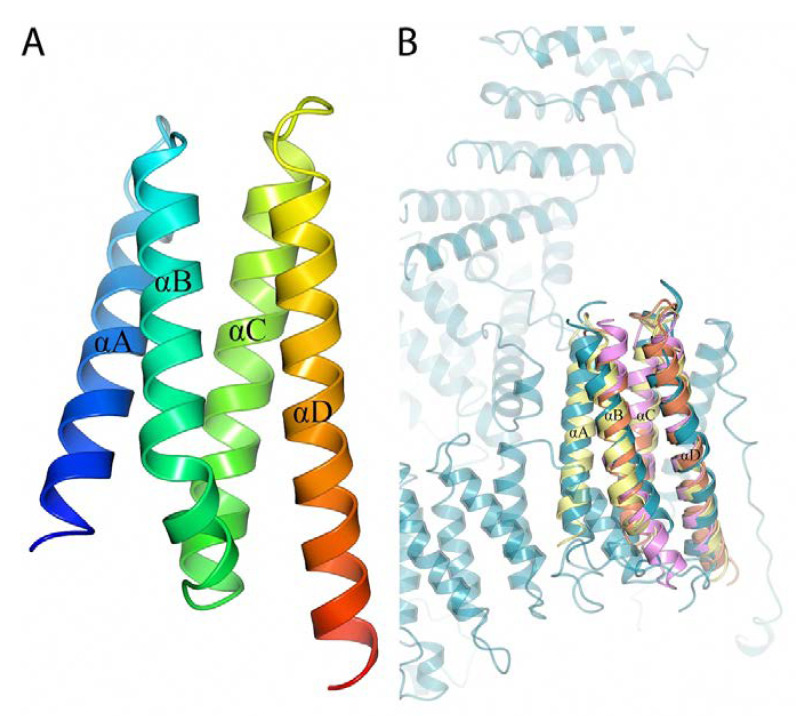
(**A**) 3D structure of *B. burgdorferi* BBP25 (OrfC) predicted with AlphaFold. The four α-helices are labeled from αA to αD. A rainbow color scheme was used starting from blue at the N-terminus and gradually switching to red toward the C-terminus. (**B**) Superimposed 3D structure of predicted *B. burgdorferi* BBP25 (yellow) with the crystal structures of *L. pneumophilia* Lem22 (orange, PDB ID 5WD8), *A. thaliana* C-terminal domain of Bcl-2-associated athanogene (pink, PDB ID 4HWH) and *H. sapiens* Fanconi anemia group D2 protein (cyan, PDB ID 6VAE).

BBA14 and the antitoxin epsilon from *S. pyogenes* showed high overall fold similarity (C^α^ root-mean-square deviation of 2.90 Ǻ) but only 20% amino acid sequence identity. Although there is a noticeable structural variability at the loop regions, the α-helices that in the *S. pyogenes* antitoxin are responsible for the interaction with the toxin are largely conserved [40]. In case BBA14 or any other PFam143 member is an antitoxin, the low sequence similarity with the antitoxin epsilon from *S. pyogenes* is not unusual because antitoxins are characterized by substantial sequence and structural diversity [42]. The same applies for toxin molecules, which can show poor sequence similarity that makes it difficult to predict the toxin gene, if any, in *B. burgdorferi*, although usually the toxins are cotranscribed with antitoxins from an operon [41]. In the case of the previously mentioned operon *ABCD* found on cp32 and lp56, but incomplete on lp54 because it lacks *orfC*, the function for OrfC is still unknown, but the structure predicted with AlphaFold reveals a four-helical bundle protein (Figure 5A).

PDBeFold [38] search against the available protein structures in the PDB shows structural similarity to effector protein Lem22 from *Legionella pneumophilia*, the C-terminal domain of Bcl-2-associated athanogene (BAG) from *Arabidopsis thaliana* and C-terminal helical repeat domain in Fanconi anemia group D2 protein from *Homo sapiens* (C^α^ root-mean-square deviation 2.24 Ǻ, 2.51 Ǻ and 2.38 Ǻ respectively) (Figure 5B) [43,44]. Lem22 by the help of type IV secretion system (T4SS) is translocated into the host cell where it interferes with the host but the exact function is still unknown [43]. The C-terminal domain of BAG protein acts as a nucleotide-exchange factor for stress-induced chaperone Hsp70/Hsc70 [44]. In turn, Fanconi anemia group D2 protein is important for chromosomal stability and DNA repair [45,46]. But because the sequence similarity to these proteins is very low (7; 9 and 11% accordingly), whether the structural similarity is also reflected in any functional similarity remains to be determined.

To date, no toxin-antitoxin system has been identified in *B. burgdorferi*, although in many other prokaryotes toxin-antitoxin systems are very abundant and serves as a mechanism to maintain plasmid content during cell growth [10,41,47]. Moreover, in *B. burgdorferi* the putative role of such a toxin-antitoxin system could be responsible for maintenance and inheritance of plasmids through subsequent generations.

Regarding the potential function of BBA14, it should be noted that previous studies have shown that deleting a region encompassing *bba07* to *bba14* on lp54 had no significant effect on the infectious cycle of *B. burgdorferi*; although the possible compensatory effect from the presence of paralogous proteins was not considered [48].

## 3. Materials and Methods

### 3.1. Cloning and Expression of BBA14

*bba14* (Gene ID: 11473658; locus tag BB_RS05175) was amplified by PCR from the genomic DNA of *B. burgdorferi* strain B31 using the primers 5’-CAT GCC ATG GGC CTT CCA GAA CCA TCA-3’ and 5’-GCT TGC GGC CGC TTA AGG TAT ATT TTT TGA GTA-3’ (NcoI and NotI recognition sites in the primers are underlined). The lipoprotein signal peptide coding sequence as predicted by SignalP 4.1 [49] and the segment coding for the first few unstructured residues as predicted by JPred4 [50] which together corresponds to residues 1–25, was excluded from the amplified gene. The amplified bba14 was ligated into the pETm-11 expression vector containing an N-terminal 6xHis tag followed by a tobacco etch virus (TEV) protease cleavage site. The construct coding for BBA14_26–121_ was transformed into Escherichia coli XL1-Blue, and the cells were incubated at 37 °C on LB agar plates supplemented with kanamycin. After 24 h, the obtained colonies were transferred to LB medium supplemented with kanamycin, and after another 24 h, the plasmid DNA was isolated and verified by DNA sequencing. The validated construct was transformed into E. coli BL21 (DE3), and the cells were incubated in 2xTY medium at 37 °C until the OD_600_ reached 0.8–1.0, followed by protein expression induced with 0.2 mM IPTG. The cells were further incubated for 6–8 h. After the incubation period, the cells were harvested by centrifugation.

### 3.2. Purification of Recombinant BBA14

The cells were resuspended in a solution of 300 mM NaCl, 25 mM NaH_2_PO_4_, 10 mM imidazole (pH 7.0) and 10 mM PMSF and lysed by sonication. The lysate was centrifuged at 10,000 rpm for 30 min at 4 °C. The soluble fraction was loaded onto a Ni-NTA agarose (Qiagen, Hilden, Germany) gravity-flow column. The 6xHis-tagged protein was eluted from the column in 300 mM imidazole (pH 7.0), 300 mM NaCl and 20 mM NaH_2_PO_4_. The buffer of the eluted protein was exchanged to 20 mM Tris-HCl (pH 8.0) using an Amicon centrifugal filter unit (Millipore, Burlington, MA, USA). The N-terminal 6xHis tag was removed by mixing the protein with recombinant TEV protease and incubating the mixture for 16–20 h at room temperature. The cleaved 6xHis tag and TEV protease were removed from the mixture via Ni-NTA agarose purification, and BBA14_26–121_ was collected in the flow-through fraction. The protein was further concentrated, and the buffer was exchanged to 10 mM Tris-HCl (pH 8.0) using an Amicon centrifugal filter unit.

### 3.3. Crystallization of BBA14

For crystallization, 96-well sitting drop plates were set using a Tecan Freedom EVO100 workstation (Tecan Group, Männedorf, Switzerland) by mixing 0.4 μL of protein (7 mg/mL in 10 mM Tris-HCl, pH 8.0) with 0.4 μL of precipitant using the 96-reagent sparse-matrix screens JCSG+ and Structure Screen 1&2 (Molecular Dimensions, Newmarket, UK). Elongated, rectangular crystals appeared after 3–4 months in a precipitant solution containing 0.1 M MES (pH 6.5) and 2.1 M (NH_4_)_2_SO_4_. Prior to data collection, the crystals were frozen in liquid nitrogen without cryoprotectant.

### 3.4. Data Collection and Structure Determination

Diffraction data for Se-Met *B. burgdorferi* BBA14 were collected at the MX beamline instrument BL 14.1 at Helmholtz-Zentrum, Berlin [51]. Reflections were indexed by XDS and scaled by AIMLESS from the CCP4 suite [52,53,54]. The initial phases were obtained by SHELX C/D/E [55], and the corresponding protein model was built automatically in BUCCANEER [56]. The crystal structure was improved by manual rebuilding in COOT [57]. Crystallographic refinement was performed using REFMAC5 [58]. The coordinates and the structure factors for *B. burgdorferi* BBA14 have been deposited in the Protein Data Bank with accession number 7QDV. A summary of the data collection, refinement and validation statistics for BBA14 is given in Table 1.

### 3.5. Protein Structure Prediction

AlphaFold v2.0 [33] was used to predict the 3D structures for *B. burgdorferi* PFam143 member proteins BBG25 (Gene ID: 1194100; locus tag BB_RS04620), BBA14 (Gene ID: 11473658; locus tag BB_RS05175), BBP26 (Gene ID: 1194454; locus tag BB_RS05970), BBN26 (Gene ID: 1194710; locus tag BB_RS07240), BBR26 (Gene ID: 1194541; locus tag BB_RS06395), BBQ33 (Gene ID: 1194769; locus tag BB_RS07565), BBM26 (Gene ID: 1194577; locus tag BB_RS06610), BBL26 (Gene ID: 1194679; locus tag BB_RS07025), BBO26 (Gene ID: 1194635; locus tag BB_RS06815) and BBS26 (Gene ID: 1194486; locus tag BB_RS06180). Moreover, the structures were predicted for BBA12 (Gene ID: 1194332; locus tag BB_RS05165), BBA13 (Gene ID: 1194333; locus tag BB_RS05170), BBP23 (Gene ID: 1194451; locus tag BB_RS05955), BBP24 (Gene ID: 1194452; locus tag BB_RS05960) and BBP25 (OrfC; Gene ID: 1194453; locus tag BB_RS05965). Access using Gene ID is available at https://www.ncbi.nlm.nih.gov/gene/; accessed on 9 January 2022. In all cases the full-length protein sequence was used for structure prediction but for structural analysis the N-terminal signal peptide region (hydrophobic α-helix) for BBG25, BBA14, BBP26, BBN26, BBR26, BBQ33, BBM26, BBL26, BBO26, and BBS26 was excluded. Structure prediction with AlphaFold v2.0 was performed according to the default parameters as indicated at the website (https://github.com/deepmind/alphafold/running, accessed on 21 January 2022) on AMD Ryzen Threadripper 2990WX 32-Core; 128 GB RAM; 4 x NVIDIA GeForce RTX 2080, and using the full databases downloaded on 2021-09-25. For further structural analysis only the predicted structures with the highest confidence were used (as ranked by using LDDT (pLDDT) scores).

## 4. Conclusions

BBA14 is one of more than 130 lipoproteins found in *B. burgdorferi*. Many of these lipoproteins are known to interact with the host and fight against its immune system; hence, they are important in Lyme disease pathogenesis. BBA14 has some differences from the other paralogous proteins belonging to PFam143, which may also indicate functional diversification. In contrast to other PFam143 members located on cp32 and lp56, *bba14* found on lp54 is not a part of a characteristic *ABCD* operon. BBA14 and another PFam143 member, BBG25, show less sequence similarity with the other PFam143 members and in contrast to the other eight PFam143 members, which are most likely cytosolic membrane proteins, BBA14 and BBG25 are lipoproteins attached to the outer membrane of *B. burgdorferi*.

The crystal structure of BBA14 revealed that the overall fold of the protein is similar to antitoxin epsilon from *S. pyogenes.* Structure prediction with AlphaFold, along with sequence analysis, indicated a highly conserved overall fold between the PFam143 members. This in turn can mean that if not all, then some of the PFam143 members could act as components of a potential toxin-antitoxin system in *B. burgdorferi* which could serve as a plasmid stabilization mechanism in a growing bacterial population, although further research is needed to confirm this assumption.

## Figures and Tables

**Table 1:** Statistics for data and structure quality.

Dataset	BBA14
X-ray diffraction data	
PDB entry	7QDV
Beamline	14.1 BESSY II, Helmholtz-Zentrum, Berlin
Space group	P 4_1_2_1_2
*a*, *b*, *c* (Å)	36.4, 36.4, 129.9
*α*, *β*, *γ* (°)	90.0, 90.0, 90.0
Wavelength (Å)	0.97961
Resolution (Å)	35.08–1.90
Highest resolution bin (Å)	1.95–1.90
No. of reflections	182,843
No. of unique reflections	7534
Completeness (%)	99.8 (100.0)
R_merge_	0.06 (0.38)
CC_1/2_	1.00 (0.99)
*I/σ* (*I*)	32.8 (9.3)
Multiplicity	24.3 (25.7)
Refinement	
R_work_	0.189 (0.234)
R_free_	0.245 (0.292)
Average B-factor (Å^2^)	
Overall	43.0
From Wilson plot	27.9
No. of atoms	
Protein	774
Water	36
RMS deviations from ideal	
Bond lengths (Å)	0.009
Bond angles (^o^)	1.565
Ramachandran outliers (%)	
Residues in most favored regions (%)	97.87
Residues in allowed regions (%)	2.13
Outliers (%)	0.00

Note: values in parentheses are for the highest resolution bin.

## Data Availability

The datasets generated during and/or analysed during the current study are available from the corresponding author on reasonable request.
